# Hot Water for Inflamed Mucous Surface

**Published:** 1885-02

**Authors:** 


### HOT WATER FOR INFLAMED MUCOUS SURFACE.

Dr. George R. Shepherd, of Hartford, Conn., adds his testimony to that of many others by saying in the *Medical Record:* "I have used hot water as a gargle for the past six or eight years, having been led to do so from seeing its beneficial effects in gynecology. In acute pharyngitis and tonsilitis, if properly used at the commencement of the attack, it constitutes one of our most effective remedies, being frequently promptly curative. If used later in the disease or in chronic cases, it is always beneficial, though perhaps not so immediately curative. To be of service it should be used in considerable quantity (half pint or pint) at a time, and just as hot as the throat will tolerate. I have seen many cases of acute disease thus aborted, and can commend the method with great confidence. I believe it may be taken as an established fact that in the treatment of inflammations generally, and those of the mucous membranes in particular, moist heat is of service, and in most cases hot water is preferable to steam. All are familiar with its use in ophthalmia and conjunctivitis, as also in inflammation of the external and middle ear, and I feel confident that those who employ it for that most annoying of all slight troubles to prescribe for, viz., a cold in the head or acute coryza, will seldom think of using the irritating drugs mentioned in the books, nor of inducing a complete anæsthesia with chloroform in preference to the hot-water douche."

